# Basal and Calcium-Stimulated Procalcitonin for the Diagnosis of Medullary Thyroid Cancers: Lights and Shadows

**DOI:** 10.3389/fendo.2021.754565

**Published:** 2021-10-13

**Authors:** Simona Censi, Marta Di Stefano, Andrea Repaci, Teresa Benvenuti, Jacopo Manso, Uberto Pagotto, Maurizio Iacobone, Susi Barollo, Loris Bertazza, Francesca Galuppini, Clara Benna, Gianmaria Pennelli, Mario Plebani, Diego Faggian, Carla Colombo, Laura Fugazzola, Caterina Mian

**Affiliations:** ^1^ Endocrinology Unit, Department of Medicine (DIMED), University of Padua, Padua, Italy; ^2^ Division of Endocrine and Metabolic Diseases, Istituto di Ricovero e Cura a Carattere Scientifico (IRCCS) Istituto Auxologico Italiano, Milan, Italy; ^3^ Department of Pathophysiology and Transplantation, University of Milan, Milan, Italy; ^4^ Division of Endocrinology and Diabetes Prevention and Care, IRCCS Azienda Ospedaliero-Universitaria di Bologna, Bologna, Italy; ^5^ Endocrine Surgery Unit, Department of Surgical, Oncological and Gastroenterological Sciences (DiSCOG), University of Padua, Padua, Italy; ^6^ Surgical Pathology and Cytopathology Unit, Department of Medicine (DIMED), University of Padua, Padua, Italy; ^7^ Surgery Unit, Department of Surgical, Oncological and Gastroenterological Sciences (DiSCOG), University of Padua, Padua, Italy; ^8^ Laboratory Medicine, Department of Medicine (DIMED), University of Padua, Padua, Italy

**Keywords:** procalcitonin, medullary thyroid carcinoma, diagnosis, markers, calcitonin

## Abstract

**Background:**

Procalcitonin (proCt) was recently proposed as an alternative or in addition to calcitonin (Ct) in medullary thyroid cancer (MTC) diagnostics.

**Methods:**

Serum basal Ct (bCt) and proCt (bproCt) levels were measured before surgery from a consecutive series of patients with (n=43) and without (n=75) MTC, retrospectively collected in Padua. Serum bproCt, bCt and stimulated proCt and Ct (sproCt and sCt) were measured in another consecutive series of 33 patients seen at three tertiary-level institutions undergoing a calcium stimulation test prior to surgery, 20 of them with a final diagnosis of MTC, and 13 with non-MTC nodular disease.

**Results:**

Median bproCt levels were higher in MTC than in non-MTC. A positive correlation was found for bproCt with bCt (P<0.01, R^2^ = 0.75), and with tumor size (P<0.01, R^2^ = 0.39). The cut-off for bproCt differentiating between MTC and non-MTC patients was >0.07 ng/ml (sensitivity: 85.7%, specificity: 98.9%, positive predictive value [PPV]: 98.2%, negative predictive value [NPV]: 90.6%, P<0.01). While bproCt was >0.07 ng/ml in 38/39 (97.4%) patients with MTC >10 mm, it was above said cut-off only in 15/23 (65.2%) patients with tumors ≤10 mm. A sproCt >0.19 ng/ml was able to identify MTC [sensitivity: 90.0%, specificity:100.0%, PPV: 100.0%, NPV: 86.7% (P<0.01)].

**Conclusions:**

Our data suggest that bproCt can be a good adjunct to Ct for MTC diagnostic purposes. In consideration of its high specificity, it can be used in combination with Ct in MTC diagnostics, particularly in the case of mildly elevated basal Ct levels.

## Introduction

Measuring calcitonin (Ct) levels is the most accurate way to detect medullary thyroid carcinoma (MTC) in patients with a nodular thyroid disease, enabling its diagnosis in earlier stages and improving the prognosis (1). Nevertheless, there are still many unsolved issues regarding the specificity of Ct assay and the interpretation of the findings, especially in cases of mildly elevated Ct levels (2). Ct is a 32-amino-acid hormone biosynthesized from the polypeptide precursor procalcitonin (proCt) [1], a 116-amino-acid prohormone. In addition to Ct (1–32), the serum of healthy subjects contains intact proCt and a number of Ct precursors that occur in concentrations twice as high as that of Ct, both in normal conditions and in MTC (3). Ct assay suffers from several pre-analytical, analytical and post-analytical pitfalls. Pre-analytically, various physiological and pathological conditions other than MTC can be associated with hypercalcitoninemia, such as male sex, smoking, drugs, thyroidal and non-thyroidal diseases (including hypergastrinemia, hypercalcemia, neuroendocrine tumors, and chronic renal failure) (2). From an analytical standpoint, the two-site/two-step fully-automated chemiluminescent immunometric (CLIA) or immunoradiometric (IRMA) assays used nowadays are more specific for Ct than older radioimmunoassays (RIA), which frequently recognized Ct precursors together with Ct. They are still prone to interferences, however. With CLIA and IRMA these interferences can come from heterophilic antibodies (3), and from the recently-described macro-calcitonin phenomenon (4). Other analytical limits concern: the short half-life of Ct, which is between 15 and 40 minutes, and varies considerably depending on its concentrations (5, 6); its variability during the day, depending on its pulsatile secretion, which is also influenced by food intake; and its instability at room temperature, at which it is rapidly degraded by serum proteases (6). This means that serum samples must be drawn in the morning after fasting overnight, placed immediately on ice and analyzed without delay. There are plenty of assay kits on the market, but they rely on different antibodies to recognize Ct, and this leads to a poor inter-method and inter-laboratory agreement for both manual and automated immunometric methods (7). When basal Ct levels are found slightly higher than the normal range, it becomes necessary to perform a confirmatory stimulation test. Here again, however, there are still no generally-agreed cut-offs for calcium-stimulated Ct, although several have been proposed (8, 9). The cytological evaluation of the fine needle aspiration (FNA) has a poor accuracy in identifying MTC (10), but its accurateness can be markedly increased by measuring Ct on washout fluids (11, 12). Indeed, Ct measurement on washout fluids has been recently introduced in the latest American Thyroid Association (ATA) MTC guidelines, that recommend for its use in case of inconclusive or MTC-suggestive cytological findings (13). However, the FNA remains an invasive procedure and it is not always easy to perform, especially in case of small nodes or multinodular goiters.

The precursor proCt was recently suggested as a potential marker of MTC, particularly in the context of suspect clinical circumstances. It has an excellent stability, with a half-life of about 20-24 hours irrespective of its concentration, and no need to be kept on ice after collection (14). All the available commercial kits also use the same antibodies, yielding similar results and making it possible to obtain standardized proCt cut-offs for MTC diagnosis and follow-up that can be shared internationally (15). As for the post-analytical phase, there is a growing body of evidence to support the use of proCt as a marker in the diagnosis and follow-up of MTC (14, 16–20). A strong correlation has also been found for basal proCt with tumor size and lymph node involvement (21). In the pre-surgical setting, three studies (21–23) have shown that both basal and pentagastrin (Pg)-stimulated proCt levels seem to be accurate in identifying MTC: however, these studies have been carried out on limited series of MTC or non-C cell nodular thyroid diseases, making difficult to define the real accuracy of this marker in term of sensitivity and specificity. Moreover, no data are yet available on proCt response to calcium stimulation test and its usefulness in MTC diagnosis.

The main aim of our study was to establish the accuracy proCt as a diagnostic marker of MTC. Secondly, in a subgroup of patients, we examined proCt responsiveness to the calcium stimulation test, and the diagnostic accuracy of stimulated proCt.

## Patients and Methods

### Patients

A serum and tissue bank has been operating at the Padua University Hospital since 2005. All patients undergoing surgery for thyroid diseases are asked for their consent to their tissue and serum samples (the latter obtained before surgery) being collected and stored for research purposes. From this bank we retrospectively and consecutively collected the sera of 43 patients who underwent thyroidectomy between January 2010 and April 2016 with a histologically-confirmed diagnosis of MTC. We also retrospectively collected serum samples (from the same bank) from 75 consecutive patients who underwent thyroidectomy between January 2017 and October 2018 for histologically-confirmed non-C-cell thyroid nodules (non-CTN). Basal proCt and Ct were measured in all these patients’ serum samples, and their clinical-pathological data were retrieved from our electronic files.

To examine the responsiveness of proCt to calcium infusion and the diagnostic value of the stimulated precursor (sproCt), a consecutive series of 33 patients (20 with and 13 without sporadic MTC; and of the latter with C-cell hyperplasia [CCH] at final histology) were enrolled. These patients were collected between January 2016 and December 2020 on a multicentric prospective basis. The three Italian institutions involved - the Azienda Ospedaliera e Università di Padova in Padua with 16 patients (hereafter Institution #1); the Istituto Auxologico Italiano Istituto di Ricovero e Cura a Carattere Scientifico (IRCCS) in Milan with 10 patients (Institution #2); and the IRCCS Azienda Ospedaliero-Universitaria di Bologna in Bologna with 7 patients (Institution #3) – had engaged in a previous collaborative study (8).

Only cases of sporadic MTC were considered, while patients carrying a germline *RET* mutation were excluded, and so were those whose *RET* germline mutational status was not known.

The calcium stimulation test included measuring basal and stimulated Ct (bCt and sCt, respectively), and proCt (bproCt and sproCt, respectively). All patients gave their informed consent to the test, and to the inclusion of the results in the present study. The study was approved by all the involved committee institutions (8). The protocol reference code number of the Padua Hospital Ethical Committee - the leader and proposing institution or the present study - was 3388. The calcium stimulation test was performed as described elsewhere (24) at all three institutions. None of the patients had clinically-evident active bacterial infections or fever at the time of their medical examination and calcium test.

All MTC patients were treated with total thyroidectomy including the central neck compartment (VI), with dissection of the involved lateral neck compartments (levels II–V) where necessary, according to the guidelines (13).

### Laboratory Assays

All three institutions measured Ct using a two-site CLIA, (Immulite2000; Siemens Diagnostics, New Jersey, USA) with an analytical sensitivity of 2 ng/L. ProCt was measured at Institutions #1 and #3 using a two-site, two-step CLIA LIAISON BRAHMS PCT II GEN (DiaSorin, Saluggia, Italy), with an analytical sensitivity of 0.04 ng/ml, and at Institution #2 using an enzyme-linked fluorescence assay (ELFA) (BioMerieux VIDAS BRAHMS PCT, Hazelhood, USA) with an analytical sensitivity of 0.05 ng/ml.

### Statistical Analyses

The Kolmogorov-Smirnov test was used to assess the normal distribution of each variable. As the variables were not distributed normally, data are reported as medians and interquartile ranges (IQR). The Mann-Whitney test for independent non-parametric data was used to analyze how proCt and Ct related to final histology (MTC or non-MTC), lymph node involvement (N0/N1), stage (I+II *versus* III+IV), and gender. The Kruskal-Wallis test for independent non-parametric data was used to analyze the relationship between bproCt and patients’ T status. Correlations between variables were studied after a logarithmic transformation for variables that were not distributed normally. Categorical variables (the relationship between dichotomized proCt and Ct values and MTC/non-MTC histology) were compared with the ϰ^2^ test. Wilcoxon’s test for paired parametric samples was used to analyze the relationship between basal and stimulated proCt values. The cut-offs affording the greatest accuracy in differentiating between MTC and non-MTC cases based on proCt values were established from the receiver operating characteristic (ROC) curves, and their sensitivity and specificity were calculated. A P-value of <0.05 was considered statistically significant.

## Results

### Patients

**Table 1** shows the clinical-pathological characteristics and biochemical data for the set of retrospectively-collected MTC. This series consists of 43 patients (22 females and 21 males; median age 61.3 years; age range 32.0-87.4 years). **Table 2** refers to the other set of retrospectively-collected patients with non-CTN disease. This series consisted of 75 patients (44 females and 31 males; median age 54.0 years; age range 18.0-83.0 years) with the following histologies: 17 follicular adenomas (FA); 20 hyperplastic goiters (HG); 18 follicular thyroid cancers (FTC); and 20 papillary thyroid cancers (PTC).

**Table 1:** Clinical features of the patients retrospectively collected from University of Padua tissue bank submitted to thyroid surgery for medullary thyroid cancer (MTC).

	Age, yrs	Basal Ct (pg/ml)	Basal proCt (ng/mL)	Histology	Cancer size (mm)	T	N	M	Stage
**Females**									
1	75	824.0	0.87	MTC	18	1	0	0	1
2	57	4000.0	11.70	MTC	30	2	1b	1	4
3	62	480.0	1.17	MTC	12	1	0	0	1
4	57	32.8	0.13	MTC	11	1	0	0	1
5	61	413.0	5.41	MTC	26	2	0	0	2
6	64	19000.0	319.00	MTC	30	3	1b	1	4
7	61	2258.0	2.76	MTC	30	2	1a	0	3
8	58	59.0	0.32	MTC	7	1	0	0	1
9	72	44.0	<0.04	MTC	10	1	0	0	1
10	37	2268.0	3.89	MTC	20	3	1a	0	3
11	68	101.0	0.35	MTC	13	1	0	0	1
12	45	83.0	0.52	MTC	10	1	0	0	1
13	43	58.4	<0.04	MTC	5	3	0	0	2
14	62	20.4	0.07	MTC	8	1	1a	0	3
15	49	267.0	0.94	MTC	18	1	0	0	1
16	62	72.0	<0.04	MTC	8	1	0	0	1
17	74	174.0	1.32	MTC	nd	–	1b	0	4
18	66	231.0	0.69	MTC	13	1	0	0	1
19	41	12.1	<0.04	MTC	5	1	0	0	1
20	53	80.0	0.24	MTC	11	1	0	0	1
21	55	493.0	0.98	MTC	22	2	0	0	2
22	64	41.3	0.6	MTC	10	1	0	0	1
**Males**									
1	75	1500.0	29.7	MTC	25	2	0	0	2
2	67	690.0	0.62	MTC	24	2	0	0	2
3	62	149.0	0.06	MTC	9	3	0	0	2
4	54	2000	53.3	MTC	5	1	1b	0	4
5	51	118	0.95	MTC	12	1	0	0	1
6	49	1274.0	9.68	MTC	4	1	1b	0	4
7	66	758.0	0.12	MTC	19	1	0	0	1
8	64	198	0.6	MTC	17	1	0	0	1
9	55	200	10.7	MTC	47	3	0	0	2
10	55	81	<0.04	MTC	4	1	0	0	1
11	76	9430	38.6	MTC	41	3	1a	0	3
12	32	654	14	MTC	42	3	1a	0	3
13	74	9660	741	MTC	51	3	1b	1	4
14	45	532	8.14	MTC	24	3	1b	0	4
15	41	1600	15.5	MTC	11	3	1b	0	4
16	73	2310	16.4	MTC	55	3	1b	0	4
17	66	2000	28.5	MTC	26	3	1b	1	4
18	48	5500	20.4	MTC	11	3	1a	0	3
19	87	390	3.39	MTC	6	1	1a	0	3
20	56	3720	6.08	MTC	24	2	0	0	2
21	73	154	0.49	MTC	28	3	1b	0	4

Ct, calcitonin; proCt, procalcitonin.

**Table 2:** Clinical features of the patients retrospectively collected from University of Padua tissue bank submitted to thyroid surgery for non-C-cell thyroid nodular disease.

	Age, yrs	Basal Ct (pg/ml)	Basal proCt (ng/mL)	Histology (mm)
**Females**				
1	40	<1	<0.04	PTC
2	29	<1	<0.04	PTC
3	47	<1	<0.04	PTC
4	37	2.3	<0.04	PTC
5	56	<1	<0.04	PTC
6	44	<1	<0.04	PTC
7	26	2.6	<0.04	PTC
8	32	4.1	<0.04	PTC
9	75	<1	<0.04	PTC
10	75	<1	<0.04	PTC
11	81	<1	<0.04	PTC
12	54	<1	<0.04	PTC
13	46	<1	<0.04	PTC
14	36	<1	<0.04	PTC
15	73	1.4	<0.04	PTC
16	55	3.6	<0.04	PTC
17	45	<1	<0.04	FTC
18	55	<1	<0.04	FTC
19	62	<1	<0.04	FTC
20	71	<1	<0.04	FTC
21	64	<1	<0.04	FTC
22	71	1.2	<0.04	FTC
23	68	9.7	<0.04	FTC
24	57	1.3	<0.04	FTC
25	42	1.1	<0.04	FA
26	55	1.1	<0.04	FA
27	53	8.9	<0.04	FA
28	57	<1	<0.04	FA
29	40	<1	<0.04	FA
30	37	<1	<0.04	FA
31	55	<1	<0.04	FA
32	51	<1	<0.04	FA
33	51	<1	<0.04	FA
34	53	<1	<0.04	HG
35	43	<1	<0.04	HG
36	56	<1	<0.04	HG
37	72	<1	<0.04	HG
38	65	<1	<0.04	HG
39	53	<1	<0.04	HG
40	53	1.7	<0.04	HG
41	44	<1	<0.04	HG
42	59	<1	<0.04	HG
43	71	4.1	<0.04	HG
44	48	7.7	<0.04	HG
**Males**				
1	29	<1	0.27	PTC
2	36	1.1	<0.04	PTC
3	78	<1	<0.04	PTC
4	67	1.1	0.05	PTC
5	58	1.1	<0.04	FTC
6	74	4.5	<0.04	FTC
7	46	2.3	<0.04	FTC
8	41	<1	<0.04	FTC
9	59	<1	<0.04	FTC
10	50	1.3	<0.04	FTC
11	83	4.7	<0.04	FTC
12	59	8.2	<0.04	FTC
13	70	2.7	<0.04	FTC
14	49	<1	<0.04	FTC
15	19	<1	<0.04	FA
16	74	<1	<0.04	FA
17	36	<1	<0.04	FA
18	43	1.2	<0.04	FA
19	53	3.2	<0.04	FA
20	58	5.4	<0.04	FA
21	69	<1	<0.04	FA
22	55	1.8	<0.04	FA
23	45	6.4	<0.04	HG
24	65	3.3	<0.04	HG
25	62	17.5	<0.04	HG
26	76	<1	<0.04	HG
27	65	<1	<0.04	HG
28	51	5.4	<0.04	HG
29	53	1.4	<0.04	HG
30	18	1.5	<0.04	HG
31	42	4.4	<0.04	HG

Ct, calcitonin; FA, follicular adenoma; FTC, follicular thyroid cancer; HG, hyperplastic goiter; MTC, medullary thyroid cancer; proCt, procalcitonin; PTC, papillary thyroid cancer.

The prospective and multicentric series of patients who had a calcium stimulation test included 20 patients with MTC (14 females and 6 males; median age 59.5 years; age range 27.7-73.0 years; **Table 3**), and 13 patients without MTC (7 females and 6 males; median age 58.0 years; age range 44.8-72.0 years; **Table 4**).

**Table 3:** Clinical features of the patients submitted to calcium-stimulation test with a final histological diagnosis of MTC.

	Age, yrs	Origin	Ct, pg/ml		proCt, ng/mL		Cancer size (mm)	T	N	M	Stage
			Basal	Peak	Basal	Peak					
**Females**											
1	62	#1	91.5	736	1.13	1.63	12	1	0	0	1
2	58	#1	39.3	488	0.32	0.46	11	1	0	0	1
3	57	#1	7720	19700	20.5	21.1	70	3	0	0	2
4	50	#1	126	175	2.33	2.5	12	1	1a	0	3
5	64	#1	49.2	974	0.38	0.52	12	1	0	0	1
6	52	#1	25.7	1010	0.31	1.59	6	1	0	0	1
7	52	#1	83.1	235	0.64	0.79	10	1	0	0	1
8	61	#1	35.3	366	0.25	0.61	11	1	0	0	1
9	27	#1	68.2	434	0.24	0.64	7	1	0	0	1
10	52	#1	61.6	538	0.26	0.37	8	1	0	0	1
11	64	#2	89.2	2707	0.77	2.36	7	1	0	0	1
12	68	#2	222	1251	1.18	1.63	11	1	0	0	1
13	66	#3	11.6	118	0.04	0.07	7	1	0	0	1
14	70	#3	53.7	678	0.12	0.4	4	1	0	0	1
**Males**											
1	46	#1	27.7	1020	0.28	0.51	17	1	0	0	1
2	73	#1	19.6	71.5	0.1	0.1	7	1	0	0	1
3	35	#2	126	1485	0.51	0.84	9	1	0	0	1
4	54	#2	23.8	665	0.19	0.29	16	1	0	0	1
5	66	#2	47	1357	0.05	0.71	12	1	0	0	1
6	69	#3	69.3	860	0.09	0.2	4	1	0	0	1

#1 Padua; #2 Milan; #3 Bologna; Ct, calcitonin; proCt, procalcitonin.

**Table 4:** Clinical features of the patients submitted to calcium-stimulation test with a final histological diagnosis negative for MTC.

	Age, yrs	Origin	Ct, pg/ml		proCt, ng/mL		Histology (mm)
			Basal	Peak	Basal	Peak	
**Females**							
1	47	#2	11.6	623	0.05	0.12	FA+CCH
2	57	#2	15.7	279	0.05	0.05	GIA+ CCH
3	64	#2	27.7	296	0.05	0.08	GIA+CCH
4	69	#2	27.8	494	0.05	0.1	PTC
5	51	#2	8.9	188	0.05	0.07	PTC
6	59	#3	28.9	891	0.07	0.14	GIA+ CCH
7	72	#3	23.8	338	0.07	0.11	HG+ CCH
**Males**							
1	54	#1	7.6	84.7	<0.04	0.07	FA
2	58	#1	19.4	238	0.05	0.12	HG+CCH
3	45	#1	18.4	157	<0.04	0.06	HG+ CCH
4	67	#1	22.4	414	<0.04	0.1	HG+CCH
5	57	#2	18.3	706	0.05	0.08	PTC
6	65	#3	16	341	0.06	0.19	HG+CCH

CCH, C-cells-hyperplasia; Ct, calcitonin; FA, follicular adenoma; HG, hyperplastic goiter; MTC, medullary thyroid cancer; proCt, procalcitonin; PTC, papillary thyroid cancer.

Our series as a whole included 63 cases of sporadic MTC and 88 patients without MTC. Among the MTC patients, there were 36 females and 27 males, with a median age of 61.3 years (range 27.7-87.4 years). The size (and consequent T stage) of one patient’s MTC was not available, while the other cancers were a median 12 mm in size (range: 4-70 mm), and 40/62 (64.5%) were T1, 7/62 (11.3%) were T2, and 15/62 (24.2%) were T3. There were 44/63 (69.8%) patients classified as N0, 8/63 (12.7%) as N1a, and 11/63 (17.5%) as N1b. Thirty-five (55.5%) of the 63 patients were in stage I, 9/63 (14.3%) in stage II, 8/63 (12.7%) in stage III, and 11/63 (17.5%) in stage IV at the time of their diagnosis. In the non-MTC group, 51 were females and 37 were males, with a median age of 55 years (range: 18-0-83.0 years).

### Basal proCt

**Figure 1:**
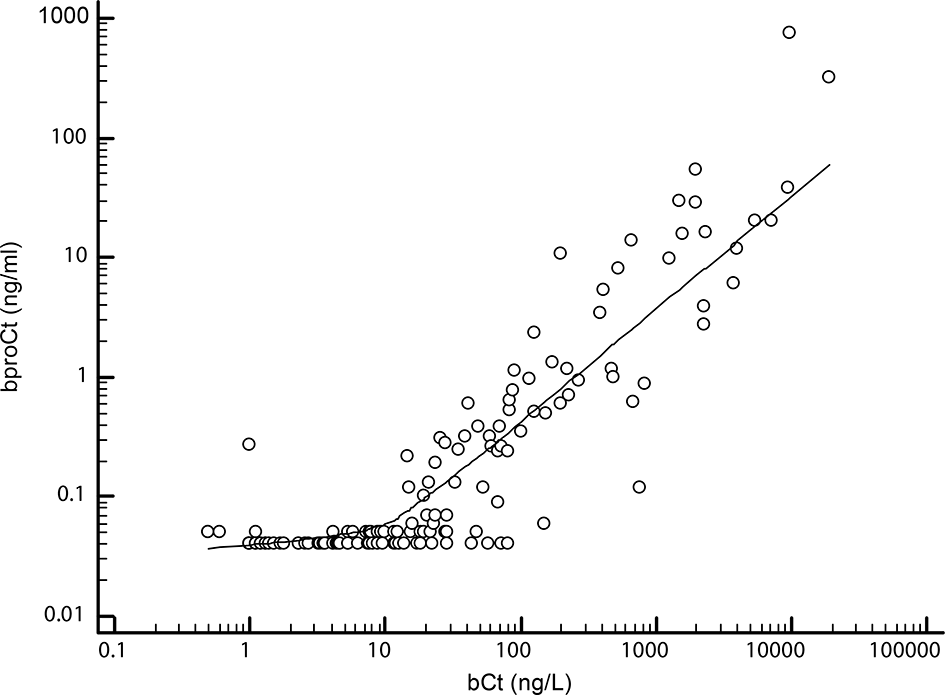
Correlation between basal procalcitonin (bproCt) and basal calcitonin (bCt) (P < 0.01, R^2^ = 0.75).

The bproCt values were significantly higher in MTC than in non-MTC patients (0.64 ng/ml, IQR 0.24-5.91 ng/ml in MTC patients *versus* 0.04 ng/ml, IQR 0-04-0.04 ng/ml in non-MTC patients, P<0.01). A strong positive correlation was found between bproCt and bCt (P<0.01, R^2^ = 0.75) (**Figure 1**).

A positive correlation also emerged between tumor size and both bproCt levels (P<0.01, R^2^ = 0.39), and bCt (P<0.01, R^2^ = 0.43). Median bproCt values paralleled the T stages, being 0.35 ng/ml (IQR 0.18-0.89 ng/ml) in T1, 5.41 ng/ml (IQR 1.43-10.29 ng/ml) in T2, and 15.9 ng/ml (IQR 6.02-33.56 ng/ml) in T3 (P<0.01).

Median bproCt levels were higher in patients with positive lymph nodes at diagnosis (N1a+N1b) than in N0 patients (11.70 ng/ml, IQR 2.92-26.48 ng/ml in N1 patients *versus* 0.35 ng/ml, IQR 0.12-0.92 ng/ml in N0 patients, P<0.01), and similar data emerged for Ct levels (2000.00 ng/L, IQR 425.50-3577.50 ng/L in N1 patients *versus* 83.00 ng/L, IQR 45.30-228.75 ng/L in N0 patients, P<0.01).

There was also an association between higher tumor stages at diagnosis and higher median bproCt levels, which were 0.34 ng/ml (IQR 0.12-0.91 ng/ml) in lower stages (stage I+II) *versus* 11.70 ng/ml (IQR 2.92-26.48 ng/ml) in higher stages (III+IV) (P<0.01). This association was found for median Ct levels too, which were 80.50 ng/L (IQR 41.30-222.00 ng/L) in lower stages (I+II) and 2000.00 ng/L (IQR 425.50-3577.50 ng/L) in higher stages (III+IV) (P<0.01).

There were no gender-related differences in bproCt levels in the series as a whole (P=0.26), or among the non-MTC patients (P=1.00), while there was a trend towards higher median bproCt levels in men than in women among the MTC patients (3.39 ng/ml, IQR 0.21-16.18 ng/ml in men and 0.56 ng/ml, IQR 0.24-1.18 ng/ml in women, P=0.06).

**Figure 2:**
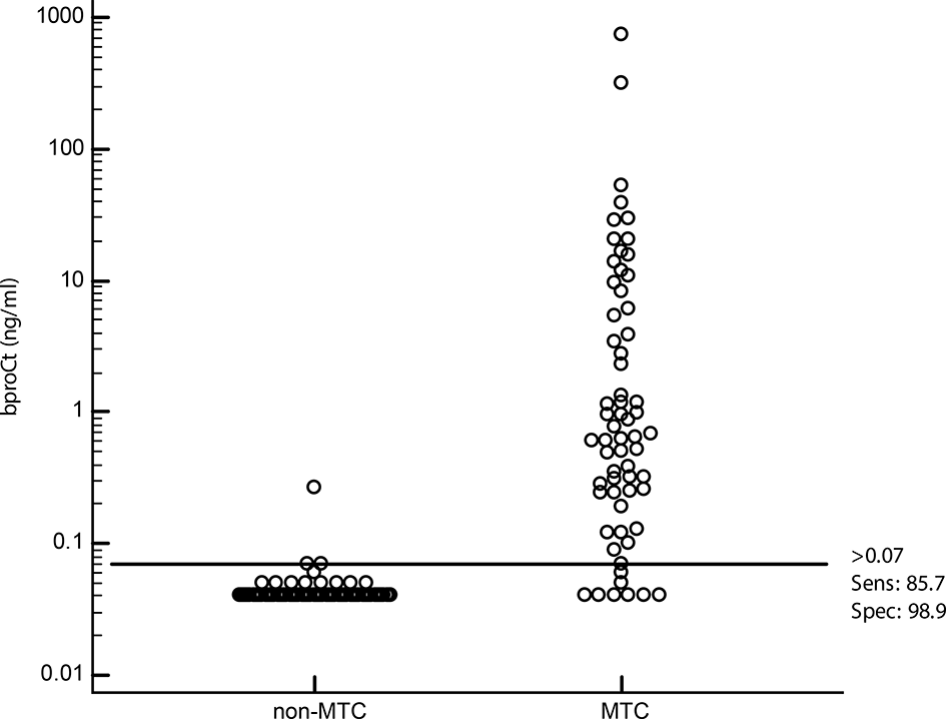
Dot plot diagram showing the accuracy of the proposed cut-off for basal procalcitonin (bproCt) in the diagnosis of MTC in patients with nodular thyroid disease.

On ROC curve analysis, the best cut-off for bproCt for differentiating between MTC and non-MTC patients was >0.07 ng/ml (sensitivity 85.7%, specificity 98.9%, positive predictive value (PPV) 98.2%, negative predictive value (NPV) 90.6%, area under the ROC curve (AUC) 0.942, and P<0.01) (**Table 5**). Only 1 of the 88 (1.1%) patients without MTC had a basal proCt >0.07 ng/ml (patient No. 1 [M] in **Table 2** and **Figure 2**). This was a 29-year-old man with a histological diagnosis of PTC, bproCt 0.27 ng/ml, and undetectable bCt: at the time of blood sampling, there was no sepsis and the histological review confirmed the absence of a C-cell disease.

**Table 5:** Basal procalcitonin (proCt) and stimulated proCt accuracies in the identification of medullary thyroid cancer (MTC) patients in our series.

Basal proCt					
	≤0.07 ng/ml	>0.07 ng/ml			
Non-MTC (N)	87	1	88	**Sensitivity (%)**	85.7
MTC (N)	9	54	63	**Specificity (%)**	98.9
Total (N)	96	55	151	**PPV (%)**	98.2
				**NPV (%)**	90.6
**Stimulated proCt**					
	**≤0.19 ng/ml**	**>0.19 ng/ml**			
Non-MTC (N)	13	0	13	**Sensitivity (%)**	90.0
MTC (N)	2	18	20	**Specificity (%)**	100.0
Total (N)	15	18	33	**PPV (%)**	100.0
				**NPV (%)**	86.7

Nine (14.3%) of the 63 patients with sporadic MTC had bproCt ≤0.07 ng/ml and MTC was confirmed at histology (patients Nos. 9, 13, 14, 16, 19 [F] and 3 and 10 [M] in **Table 1** and patients Nos. 13 [F] and 5 [M] in **Table 3**). All but one of these patients had a micro-MTC (≤10 mm). One patient (No. 14 [F] in **Table 1**) with a micro-MTC, but N1a disease, had borderline bproCt (=0.07 ng/ml) and only moderately elevated Ct (20.4 ng/L).

On ROC curve analysis, the bproCt threshold that best predicted lymph node positivity was >1.18 ng/ml (sensitivity 89.5%, specificity 88.4%, PPV 77.3% and NPV 95%, AUC 0.880, and P<0.01).

Since the bproCt cut-off was not very reliable in patients with micro-MTC (≤10 mm), we separately analyzed its performance in this subgroup of MTC. Among the 62/63 cases of MTC of known size, 23/62 (37.1%) were micro-MTC, and 39/62 (62.9%) were larger. While bproCt was >0.07 ng/ml in 38/39 (97.4%) patients with larger MTC (all except patient No. 5 [M] in **Table 3**), bproCt levels were only consistent with MTC in 15/23 (65.2%) patients with a tumor <10 mm in size.

### Stimulated proCt

Wilcoxon’s test for paired samples showed a significant difference between bproCt and sproCt levels, the latter being higher (sproCt: 0.1 ng/ml, IQR 0.05-0.45 ng/ml; bproCt: 0.05 ng/ml, IQR 0.05-0.25 ng/ml) (P<0.01), which would suggest a proCt response to calcium stimulation. In the MTC group, median bproCt was 0.29 ng/ml (IQR 0.16-0.71 ng/ml), and median sproCt was 0.58 ng/ml (IQR 0.39-1.61 ng/ml) (P<0.0001), while in the non-MTC group median bproCt was 0.05 ng/ml (IQR 0.05-0.06 ng/ml) and median sproCt was 0.1 ng/ml (IQR 0.07-0.12) (P=0.0005).

A positive correlation was found between sproCt and sCt (P<0.01, R^2^ = 0.46). Tumor size also showed a weak positive correlation with sproCt (P<0.01, R^2^ = 0.38), and with sCt (P<0.01, R^2^ = 0.33). The median Δ (the difference between the basal and stimulated proCt levels) was higher in MTC than in non-MTC patients, being Δ 0.25 ng/ml, IQR 0.14-0.44 ng/ml in the former, and Δ 0.04 ng/ml, IQR 0.03-0.07 ng/ml in the latter (P<0.01).

No gender-related differences in sproCt levels emerged, neither for the series as a whole (P=0.96), nor among non-MTC (P=0.62) or MTC patients (P=0.11).

Finally, the best sproCt cut-off for differentiating between MTC and non-MTC patients was >0.19 ng/ml (sensitivity 90.0%, specificity 100%, PPV 100%, NPV 86.7%, AUC 0.938, P<0.01) (**Table 5**). None of the non-MTC patients’ sproCt peaked over this cut-off, while 2/20 (10.0%) MTC patients (Nos. 13 [F] and 2 [M] in **Table 3**) had levels below this cut-off. Intriguingly, the latter patient showed no Ct response to stimulation either.

### Combination of proCt and Ct

We applied recently-established bCt and sCt gender-tailored cut-offs in clinical practice in a multicentric Italian study involving our institutions (8). The bCt and sCt cut-offs consistent with MTC were respectively >30 ng/L and >79 ng/L in females, and >34 ng/L and >466 ng/L in males. Applying the above-mentioned cut-offs to the present series of retrospective and prospective cases, the performance of bCt in identifying MTC reached a sensitivity of 92.6%, a specificity of 100%, a PPV of 100%, and a NPV of 88.8%. Fifty-six out of 63 (88.9%) MTC patients had bCt levels consistent with MTC, while 7 (11.1%) did not (**Tables 1**, **3**).

Focusing on the 19 patients with a bCt in the “grey” zone, i.e. >10 ng/ml but <30 (in females) or <34 (in males), 7 had MTC and 12 had non-MTC nodular disease. Among those with MTC, bproCt was able to identify MTC in 4/7 patients (57.1%). More importantly, it was able to rule out MTC in all 12 non-MTC patients since none of them had a bproCt >0.07 ng/ml. In this subgroup of patients, these data outlined a sensitivity of 57.1%, a specificity of 100%, a PPV of 100% and a NPV of 80.0%. The false negatives for bproCt in this group thus amounted to 42.9%. The 3 patients not identified by their bproCt levels were patients Nos. 14 and 19 (F) in **Table 1** and No. 13 (F) in **Table 3**. The latter underwent a calcium stimulation test in the present project, which identified her as a case of MTC with Ct peaking as high as 118 ng/L. We checked our electronic files to see if the other two had undergone a calcium stimulation test, and found that patient No. 14 had been tested and peaked as high as 286 ng/L, while patient No. 19 had not been tested prior to surgery. In short, the calcium stimulation test - when performed - was able to identify patients overlooked at the baseline biochemical steps.

Thirty-nine patients, from the whole series, had a bCt between 10 and 100 ng/L, and 12 of them (30.7%) had non-CTN disease, while 27 (69.2%) had MTC. None of the former 12 patients had bproCt levels >0.07 ng/ml, while the cut-off identified MTC in 19 (70.4%) of the other 27 patients, reaching a sensitivity of 70.4%, a specificity of 100.0%, a PPV of 100.0%, and a NPV of 40.0%, so the false negative rate for bproCt was 29.6%.

Remarkably, among the patients who had a calcium stimulation test, sCt was able to identify MTC in all but one case (patient No. 2 [M] in **Table 3**) using the reference cut-offs (8); and this patient’s bproCt was indicative of MTC (0.1 ng/ml).

## Discussion

Before it can be considered as a tumor marker for MTC, bproCt has to demonstrate a good sensitivity and specificity, and its correlation with tumor burden to confirm its prognostic utility. Machens et al. addressed the latter issue in a large series of MTC, finding a similar diagnostic accuracy for bproCt and bCt as regards their correlation with cancer size, extrathyroidal extension, lymph node involvement and distant metastases (21). We confirmed their results in our series, finding a similarly robust correlation between bproCt and bCt levels (R^2^ = 0.75 and R^2^ = 0.88, respectively), and also confirming the correlation with tumor size, and the presence of higher median proCt levels in patients with lymph node involvement than in those with negative nodes. Higher bproCt levels were associated with higher T and disease stages, confirming the relationship between bproCt at diagnosis and extent of disease (21).

Our study was designed primarily to test the sensitivity and specificity of bproCt in identifying MTC, so we analyzed its performance in a large series of histologically-proven MTC and non-MTC patients. It is important to emphasize that the primary aim was not to assess the role of proCt as a screening marker in the diagnostic work-up of thyroid nodes. It is well known that current guidelines are neither for nor against searching for MTC in such a clinical scenario (25). Based on the cut-off established from a ROC curve analysis, bproCt showed a high specificity. Paralleling the results obtained by Giovanella et al. (22, 23) in a limited number of MTC, we found bproCt elevated only in cases of MTC, with the exception of one patient in our series whose higher bproCt levels remain unexplained, since his bCt levels were negative, and Ct-negative MTC is very rare (6).

A tumor marker highly specific for MTC can be effective in certain clinical contexts. Patients with a slightly elevated bCt (between 10 and 100 ng/L) and nodular thyroid disease are those in whom diagnosing MTC (often a micro-MTC) is more challenging. In cases with Ct values in the grey zone, some authors suggest a wait-and-see strategy to avoid overtreatment (26). Alternatively, a calcium stimulation test could be performed in such cases, though some authors question the usefulness of this approach. In a recent paper by Fugazzola et al, we found that it was only by combining bCt with sCt that all cases of MTC (all T1) could be identified correctly in both genders (8). On the other hand, Niederle et al. reported that, with modern highly-sensitive Ct assays, considering sCt after calcium stimulation did not improve the accuracy of bCt alone for the diagnosis of MTC. That said, a far from negligible part of their micro-MTC patients had bCt levels overlapping those of CCH patients (9). In fact, it is worth emphasizing that the natural history of micro-MTC remains largely unknown.

Our data demonstrate that, especially in patients with slightly elevated Ct levels, a positive bproCt finding based on our cut-off (0.07 ng/ml) is highly accurate (with a specificity and PPV around 100%) in confirming MTC. Set against its high specificity, it has a sensitivity and NPV that remains lower than for bCt. Especially among patients with moderately high Ct levels, bproCt often fails to identify a case of MTC: the false negative rate for our bproCt cut-off was 14.3% in our whole series of MTC, and rose to 29.6% among patients with Ct levels between 10 and 100 ng/L, and to as high as 42.9% among those with Ct levels between 10 ng/L and the gender-oriented cut-offs suggested in the literature (8). All but one of our MTC patients not identified by bproCt had a micro-MTC, suggesting that bproCt is not sensitive enough to identify MTC in patients with a low burden of disease. On further investigating this issue, we found that our bproCt cut-off could identify MTC in all except one of the patients with MTC larger than 10 mm, but failed in 34.8% of the cases of micro-MTC.

To the best of our knowledge, this is the first report demonstrating that bproCt levels rise following a calcium stimulation test. Giovanella et al. previously showed that proCt levels increased after Pg stimulation only in patients carrying MTC (23). Conversely, a calcium response was also seen in the non-MTC group in our series, though it was fainter than in the MTC group. Finding the median increase in proCt higher in cases of MTC than in non-MTC patients enabled us to identify a possible cut-off for calcium-stimulated proCt as well, which was >0.19 ng/ml. It is worth noting (although our series was small) that this cut-off achieved a 100% specificity and PPV. Like bproCt, sproCt was always able to rule out MTC, but (like basal proCt levels) it failed to identify roughly one in ten cases of MTC, confirming its limited sensitivity and NPV. Based on the results of our study, we propose to use bproCt in the context of slightly elevated Ct levels, and not for patient screening in cases of nodular thyroid disease because of its low sensitivity. This means, again judging from our findings, that the risk of misdiagnosing a thyroid nodule as MTC on the grounds of elevated bproCt levels is near zero.

Another issue regarding proCt concerns the fact that Ct levels obtained with different commercial assays are not comparable, preventing us from establishing internationally standardized Ct cut-offs, whereas all proCt kits employ the same antibodies, potentially overcoming this problem. The Brahms Kryptor (Thermo Fisher Scientific Clinical Diagnostics, Hennigsdorf, Germany) is considered the reference method for measuring proCt, but previous reports confirmed its correlation and concordance with other proCt assay kits, including the two methods used for the patients discussed in the present report (27, 28). Both these latter assays have shown a strong correlation and concordance with the reference method, but their analytical sensitivity differs, being 0.04 ng/ml for the CLIA LIAISON kit, and 0.05 ng/ml for the BioMerieux VIDAS ELFA kit. Such a small difference in the very low range is unlikely to have significantly affected the results in the present series, however. Interestingly, the lowest proCt level found among the sporadic MTC cases described by Machens et al, who used the Brahms Kryptor assay kit, was 0.07 ng/ml (in a case of multifocal N0 micro-MTC) (21). This compares well with our present data indicating that bproCt levels >0.07 ng/ml are consistent with MTC, and pointing to the feasibility of finding an international consensus on the cut-offs for bproCt in MTC. This will demand studies on larger and possibly multicentric series (given the rarity of the disease), however.

A remarkable difference between the use of proCt and Ct cut-offs lies in that we did not find any significant gender-related difference in either basal or stimulated levels of the former. These preliminary data suggest that proCt could further simplify MTC diagnostics, possibly enabling the adoption of a universal cut-off regardless of gender.

Our study has some limitations that we would like to underscore. First, our series did not include patients with Ct-negative MTC, a rare but nonetheless possible MTC entity for which it could be useful to analyze the usefulness of proCt testing. Another possible limitation of the present study could lie in the scarce number of CCH, which prevented us from analyzing proCt behavior specifically in this histological setting, which is often associated with moderately elevated Ct levels. We hope to be able to increase the size of the present series, also involving other national and international institutions interested in gaining a better understanding of proCt diagnostic value. Another possible drawback derives from the fact that we pooled together two series of patients: the first one was a consecutive and retrospective series from a single institution and the second one was a consecutive and prospective series from three different institutions. Due to the rarity of the disease, this study design allowed us to increase the number of histologically-proven MTC, to precisely calculate proCt test accuracy. Lastly, a potential pitfall of considering proCt concerns the risk of a septic status interfering with the test results. In our series, we only included patients not carrying any infections - to judge from their clinical examination and medical history. That said, we cannot rule out the possibility of other situations giving rise to higher proCt levels, such as asymptomatic systemic bacterial infections, or some non-infectious conditions unknown to or not mentioned by patients (such as previous traumas, burns, or neuroendocrine neoplasms) (27). It could be of particular interest to look for such conditions, for example, in the one patient in our series with inexplicably high bproCt and undetectable bCt levels, and no MTC at histology, as this would further support the need to combine Ct and proCt measurements.

In conclusion, our data suggest that bproCt can be a good adjunct to Ct for MTC diagnostic purposes, and particularly useful in the case of slightly elevated basal Ct levels. Higher than normal bproCt levels can pinpoint cases of MTC among patients with nodular thyroid disease. In a real-life context, as suggested by a recent Cochrane review the prevalence of MTC is very low (around 0.32% in thyroid nodules), contributing to a very low PPV for bCt levels over the laboratory thresholds (2). In this setting, the high specificity of bproCt testing could make it very helpful for ruling out the (unlikely) possibility of clinical macro-MTC for most patients with malignant or benign thyroid nodes. Noteworthy, the availability of a single serum assessment (bproCt) instead of a multi-step, time-consuming procedure (calcium stimulation test) or a wait-and-see strategy is particularly advisable, especially in pandemic eras. These preliminary results are promising, however, and can pave the way to the search for internationally accepted proCt cut-offs.

## Data Availability Statement

The original contributions presented in the study are included in the article/supplementary material. Further inquiries can be directed to the corresponding author.

## Author Contributions

SC, CM, MF, and LF: study concept and design, data analysis and interpretation, and drafting of the manuscript. AR, TB, JM, UP, MI, SB, LB, FG, CB, GP, and CC: substantial contribution to data acquisition and interpretation and critical revision of the manuscript. MP and DF: substantial contribution to data acquisition and data analysis. All authors: final approval of the manuscript and agreement with all the aspects of the work. All authors contributed to the article and approved the submitted version.

## Funding

The authors declare that the publication fees were covered by the DIMAR (DImed and Malattie Rare) Excellence Project of the Department of Medicine, University of Padua.

## Conflict of Interest

The authors declare that the research was conducted in the absence of any commercial or financial relationships that could be construed as a potential conflict of interest.

## Publisher’s Note

All claims expressed in this article are solely those of the authors and do not necessarily represent those of their affiliated organizations, or those of the publisher, the editors and the reviewers. Any product that may be evaluated in this article, or claim that may be made by its manufacturer, is not guaranteed or endorsed by the publisher.
